# The association between type D personality, and depression and anxiety ten years after PCI

**DOI:** 10.1007/s12471-016-0860-4

**Published:** 2016-06-13

**Authors:** M.N.A. AL-Qezweny, E.M.W.J. Utens, K. Dulfer, B.A.F. Hazemeijer, R-J. van Geuns, J. Daemen, R. van Domburg

**Affiliations:** 1 Department of Cardiology, Thoraxcenter, Erasmus Medical Center Rotterdam, Rotterdam, The Netherlands; 2 Department of Child and Adolescent Psychiatry/Psychology, Erasmus MC – Sophia Children’s Hospital, Rotterdam, The Netherlands

**Keywords:** Depression, Anxiety, Coronary artery disease, PCI, Type D personality

## Abstract

**Objective:**

There are indications that type D personality and depression are associated in patients treated with percutaneous coronary intervention (PCI). However, at present it is unclear whether this relationship holds in the long term. This study’s aim was to investigate the association between type D personality at 6 months post-PCI (baseline), and depression at 10-year follow-up. A secondary aim was to test the association between type D personality at baseline and anxiety at 10-year follow-up.

**Methods:**

A cohort of surviving consecutive patients (*N* = 534) who underwent PCI between October 2001 and October 2002. Patients completed the type D personality scale (DS14) measuring type D personality at baseline, and the Hospital Anxiety and Depression Scale (HADS) measuring anxiety and depression at baseline and at 10 years post-PCI.

**Results:**

At baseline, the prevalence of type D personality was 25 % (135/534). Type D personality patients were more often depressed (42 %) than non-type D personality patients (9 %). Response rate of anxiety and depression questionnaires at 10 years was 75 %. At 10-year follow-up, 31 % of type D personality patients were depressed versus 13 % of non-type D personality patients. After adjustments, baseline type D personality remained independently associated with depression at 10 years (OR = 3.69; 95 % CI [1.89–7.19]). Type D showed a similar association with anxiety at 10 years, albeit somewhat lower (OR = 2.72; 95 % CI [1.31–5.63]).

**Conclusions:**

PCI patients with type D personality had a 3.69-fold increased risk for depression and a 2.72-fold increased risk for anxiety at 10 years of follow-up.

## Introduction

Since the introduction of drug-eluting stents in the early 2000 s, prognosis has been improved significantly [1]. Therefore, secondary outcomes such as quality of life, safety, depression and anxiety have become more important.

Personality may play a role in the mental and physical health of coronary artery disease (CAD) patients. Patients with distressed personality (type D) showed negative affectivity and social inhibition [2] and this was associated with depression and anxiety [3, 4]. Moreover type D personality corresponded with adverse cardiac outcomes in patients with CAD. One study compared high negativity/high inhibition patients (type D) with high negativity/low inhibition patients. Type D personality showed an increased risk in major adverse cardiac events such as myocardial infarction (MI), percutaneous coronary intervention (PCI), and coronary artery bypass graft (CABG) at 15 months post-PCI while high negativity/low inhibition did not [5]. These are concerning findings since the prevalence of type D personality is around 20–50 % and therefore type D is a factor that should not be ignored [6].

The prevalence of depression in patients with coronary artery disease (CAD) ranges from 25 % to 50 % [7–11]. Studies have shown an increased mortality in patients with depression. The study populations consisted of patients with CAD, CABG, PCI and heart failure [12–16]. Depressed patients may have an increased risk of adverse events after PCI [16]. Depressed patients have a poorer prognosis with higher cardiac mortality compared with non-depressed patients [17].

Because recognition of depression might be too late, since the patient is then already depressed, early recognition of type D personality could improve treatment outcomes and lead to a better prognosis, since type D was associated with an increased risk for MI, PCI, CABG and adverse prognosis [5, 18]. With timely identification of type D personality, the risks of this personality style for (cardiac) health outcomes can be communicated with the patients and, if necessary, adequate referral to a mental health professional with expertise in the field can take place.

The aim of the present study was to investigate whether type D personality, assessed at baseline, is associated with depression, not only at one year post-PCI, but also at 10 years after PCI. Anxiety at 10-year follow-up was also investigated as a secondary objective.

## Methods

### Participants and procedures

This was a consecutive prospective cohort registry of patients who underwent PCI between October 2001 and October 2002 (*n* = 1411). At 6 months post-PCI (referred to as baseline in the remainder of this paper) patients were asked to fill in psychological questionnaires to assess anxiety, depression (using the Hospital Anxiety and Depression Scale; HADS), and type D personality (using the Type D Scale-14, DS14). Assessment of 6 months post-PCI was chosen to ascertain a stable medical and mental condition. At 10 years, survival status was assessed using the civil registry. All surviving patients were sent a letter including the HADS questionnaire.

**Fig. 1:**
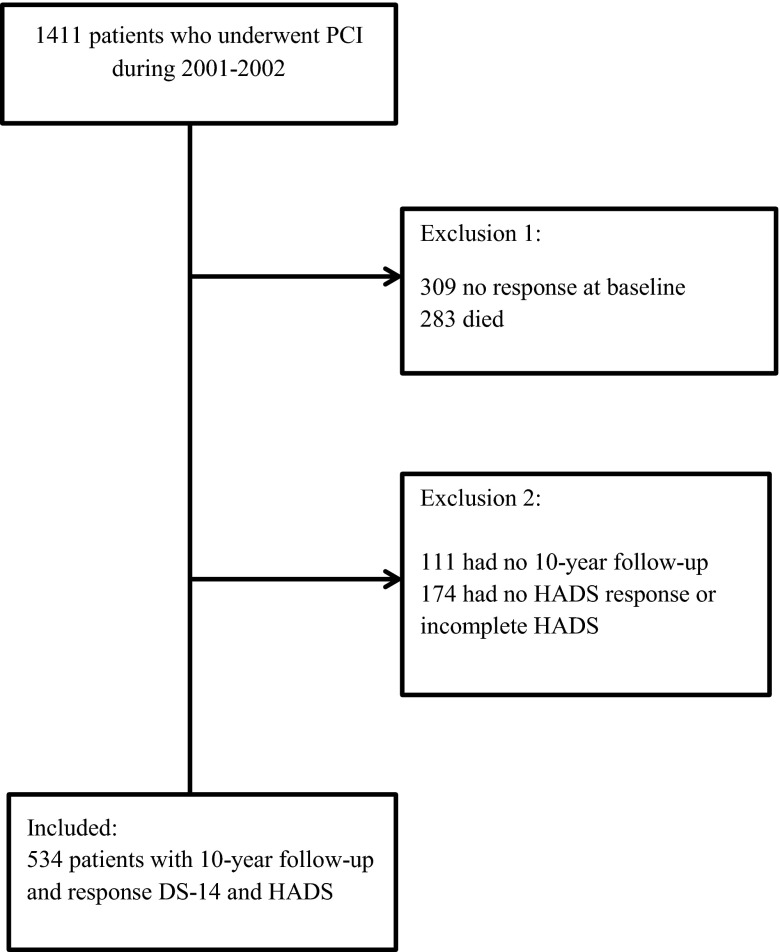
Flowchart of patient inclusion

Of the 1411 eligible patients who underwent PCI, 309 patients did not return the questionnaire at baseline. Of the remaining 1102 patients, 283 died. Of the 819 remaining patients, 534 responded to the HADS questionnaire (Fig. 1).

This study was not subject to the Dutch Medical Research Involving Human Subjects Act. Therefore, approval from the local research ethics committee to conduct this prospective follow-up study was not required at the time of enrolment. All patients consented to participation in this study by returning the questionnaire. However, in 2013 we received approval from our local medical ethics committee for this on-going PCI Registry (MEC-2013-262). The study was conducted according to the Helsinki Declaration [19].

### Sociodemographic and clinical characteristics

Sociodemographic characteristics included gender and age. Clinical characteristics included multi-vessel disease (multi-vessel disease versus single-vessel disease/no vessel disease), cardiac history (i. e., previous MI, CABG surgery, or PCI), indication for PCI (angina pectoris, unstable angina pectoris, or MI), cardiac risk factors (i. e., hypertension, hypercholesterolaemia, diabetes, family history of CAD and self-reported smoking). Information on clinical variables was prospectively collected at the time of the index-PCI/baseline and recorded in the institutional database.

### Type D personality

The DS14 was used to assess type D personality. The DS14 uses two subscales with seven items each. One scale is used to assess negative affectivity and the other one is used to assess social inhibition. Negative affectivity is a term that covers certain feelings such as ‘I often feel unhappy’ and ‘I often find myself worrying about something’. While social inhibition covers social discomfort and problems with social interactions [20]. Items can be scored from 0 (false) to 4 (true). Type D personality was identified if both subscales scored ≥ 10 points [21].

### Anxiety and depression

Anxiety and depression was assessed by completing the Dutch version of the HADS. The HADS consists of two subscales. One subscale is used to measure anxiety and the other is used to measure depression. Each subscale has seven items which can be scored from 0 to 3. A cut-off score of ≥ 8 was used to determine depression and anxiety [22].

### Statistical analysis

Patients were grouped according to type D and non-type D personality. Differences between groups were analysed using the Chi-square test for nominal variables and the Student’s t‑test for continuous variables. A multivariable logistic regression model was used to investigate the association between type D personality and depression and anxiety, respectively. Also adjustments were made for all baseline characteristics. Additionally, adjustments were made for baseline anxiety and depression. Interactions between type D personality, and anxiety and depression were investigated.

The outcomes were reported with odds ratio (OR) and their corresponding 95 % confidence interval (CI). All results were based on two-tailed tests and a *p* < 0.05 was used to indicate statistical significance. Data analysis has been done with SPSS version 21.

## Results

### Baseline characteristics

The prevalence of baseline type D personality was 25 % (135/534). The prevalence of depression at baseline was 42 % (57/135) in type D personality patients versus 9 % (35/399) in non-type D personality patients. Anxiety was also more prevalent in the type D personality patients. Furthermore, most baseline characteristics did not differ between the two groups, except for the finding that type D personality patients were more often smokers (Tab. 1).

**Tab. 1:** Baseline characteristics according to type D and non-type D

	Type D	Non-Type D	P
No. of patients	135 (25 %)	399 (75 %)	
Male	96 (71 %)	305 (77 %)	0.24
Age ± SD	59 ± 9.7	60 ± 9.6	0.19
*Clinical characteristics*			
Multivessel disease	64 (47 %)	185 (46 %)	0.83
Old MI	50 (37 %)	145 (37 %)	0.98
Old PCI	17 (13 %)	75 (19 %)	0.10
Old CABG	12 (9 %)	32 (8 %)	0.75
*Risk factors*			
Hypertension	53 (39 %)	138 (35 %)	0.33
Family history	50 (37 %)	121 (30 %)	0.15
Current smoker	57 (42 %)	123 (31 %)	0.02
Diabetes mellitus	18 (13 %)	38 (10 %)	0.21
Cholesterol	111 (82 %)	324 (81 %)	0.79
*Indication for PCI*			0.29
Stable angina	59 (44 %)	199 (50 %)	
Unstable angina	54 (40 %)	130 (33 %)	
Acute myocardial infarction	22 (16 %)	70 (18 %)	
*Medication*			
Aspirin	130 (96 %)	389 (98 %)	0.36
Calcium antagonist	72 (53 %)	189 (48 %)	0.24
Beta blocker	90 (67 %)	273 (69 %)	0.68
Oral nitrates	17 (13 %)	54 (14 %)	0.77
Diuretic	21 (16 %)	51 (13 %)	0.42
ACE inhibitor	33 (24 %)	128 (32 %)	0.09
Statin	108 (80 %)	312 (78 %)	0.69
*Psychological characteristics*			
Anxiety	73 (54 %)	61(15 %)	< 0.001
Depression	57 (42 %)	35 (9 %)	< 0.001

### Association between baseline type D personality and depression at 10 years

The prevalence of depression at 10 years of follow-up was 31 % (42/135) in type D personality patients versus 13 % (51/399) in non-type D personality patients. Univariable analysis showed a strong association between type D personality and depression at 10 years (OR = 3.08; 95 % CI [1.93–4.92]) (Tab. 2). After adjusting for baseline characteristics, type D personality showed an even stronger association with depression (OR = 3.70; 95 % CI [2.23–6.13]). In the third model, type D was adjusted for baseline characteristics and baseline depression. The OR remained the same but the CI widened (OR = 3.69; 95 % CI [1.89–7.19]). Depression at baseline was found to be a strong cofactor and therefore an interaction variable was made. The interaction between type D personality and depression at baseline proved to be significant and, therefore, the interaction was added in the multivariable model. The analysis showed that the association between type D personality and depression was stable in all models (Tab. 2).

**Tab. 2:** Univariable and multivariable analysis: association between baseline type D and depression and anxiety at 10 years

	OR[95%CI]
*Depression*	
Univariable type D personality	3.08[1.93–4.92]
Adjusted for baseline characteristics type D personality^a^	3.70[2.23–6.13]
Adjusted for baseline characteristics + baseline depression type D personality	3.69[1.89–7.19]
*Anxiety*	
Univariable type D personality	3.43[2.22–5.30]
Adjusted for baseline characteristics type D personality^a^	3.52[2.24–5.53]
Adjusted for baseline characteristics + baseline anxiety type D personality	2.72[1.31–5.63]

^a^Excluding baseline depression/baseline anxiety

### Association between baseline type D personality and anxiety at 10 years

The prevalence of anxiety at 10-year follow-up was 40 % (54/135) in type D personality patients versus 16 % (65/399) in non-type D personality patients. In univariable analysis type D personality was associated with anxiety at 10-year follow-up (OR = 3.43; 95 % CI [2.22–5.30]) (Tab. 2). After adjusting for baseline characteristics, type D personality remained associated with anxiety (OR = 3.52; 95 % [2.24–5.53]). In the third model, type D was adjusted for baseline characteristics and baseline anxiety. The OR between type D and anxiety at 10 years was still significant (OR = 2.72; 95 % CI [1.31–5.63]). Anxiety at baseline was found to be a strong cofactor and therefore an interaction variable was made. The interaction between type D personality and anxiety proved to be significant and, therefore, the interaction was added in the multivariable model.

## Discussion

Type D personality early after PCI was associated with a 3.69-fold increased risk for depression and 2.72-fold increased risk for anxiety 10 years after PCI. Earlier, our research group reported an association between type D personality and depressive symptoms at one year [3, 23]. This present study showed that this association sustained at least up to 10 years after PCI. Two other studies outside of our research group also showed an association between type D personality and depression with a follow-up period of 1 year [24, 25].

One study, however, found no relation between type D personality and the development of major depression or minor depression in 250 patients who had an acute coronary syndrome [26]. Major or minor depression in this study was measured by the DSM-IV. This raises questions whether type D personality is a personality trait or not. The limitation of that study was its small sample size. Furthermore, the HADS used in our study has been proven to be a valid questionnaire for symptom severity and case recognition of anxiety and depression disorder [22].

The patients selected for this study were a good representation of PCI patients. It should be noted that our patients were Dutch-speaking. The question can be raised whether type D personality generalises across regions and cultures. However, type D personality was shown to be generalised across cultures in a recent study [6]. Together with the findings of this study and the previous ones, it shows that type D personality is universal. Though the cross-cultural analysis of the study only extends to European and English-speaking countries, it may also extend to Asian countries [27, 28].

Despite all the evident results of the effect of type D personality, there still is a discussion concerning the validity of type D personality. Bacon et al. suggested in their commentary that while studies show that type D personality is independently associated with depression in different populations, the cut-off point of 10 was criticised [29].

The same statistical models were used to test associations between type D personality at baseline and depression and anxiety, respectively, at 10-year follow-up. Type D personality was proved to be not only associated with an increased risk for depression at 10 years of follow-up, but also with an increased risk for anxiety. Previously we reported the association between type D personality at baseline and anxiety at one year in PCI patients [4, 30]. Now we have extended our findings regarding this association to up to 10 years of follow-up.

Unfortunately, systematic data regarding the use of psychotherapy or antidepressant or antianxiety medication in our patient sample were not available. Therefore, it is unknown to what extent these factors may have influenced the present findings. Future research should also take these treatments into account.

At baseline we achieved a response rate of 79 %, which is quite reasonable. During 10 years a number of patients died or were lost to follow-up and for the remaining patients we achieved a very similar result with a response rate of 75 %. While we are of the opinion that the numbers are reasonable, we should acknowledge that this could introduce selection bias.

In summary, type D personality is a predictive tool to identify patients who have an elevated risk for depression and anxiety, 10 years after PCI. Type D personality has been shown to be a stable personality taxonomy [4]. So, presumably, a patient with type D personality will have this personality for many years or for the rest of his or her life, making it more reliable than the HADS, which is a state measure fluctuating across time and situation. The importance of these results lies in the clinical use of type D personality in identifying PCI patients with a high risk for depression and anxiety.
